# Effect of Probiotic-Assisted Eradication of *cagA*+/*vacA s1m1 Helicobacter pylori* on Intestinal Flora

**DOI:** 10.1155/2022/8607671

**Published:** 2022-04-29

**Authors:** Chenxi He, Fanting Kong, Xiukun Chai, Chunyan Zou, Xinying Zhu, Dongqiang Zhao

**Affiliations:** ^1^Department of Gastroenterology, The Second Hospital of Hebei Medical University, Shijiazhuang, 050000 Hebei Province, China; ^2^Department of Gastroenterology, Xingtai People's Hospital, Xingtai, 054000 Hebei Province, China; ^3^Department of Gastroenterology, The Third Hospital of Hebei Medical University, Shijiazhuang, 050000 Hebei Province, China

## Abstract

**Objective:**

We attempted to evaluate the effects of probiotic-assisted eradication of *cytotoxin*-*associated gene A* (*cagA*)+/*vacuolating cytotoxin A* (*vacA*) *s1m1 Helicobacter pylori* (*H. pylori*) on the intestinal flora, inflammatory factors, and clinical outcomes.

**Methods:**

A total of 180 patients with *cagA*+/*vacA s1m1* H*. pylori* were randomly divided into two groups. Group A was treated with bismuth quadruple therapy (BQT). Group B was treated with *S. boulardii* in addition to BQT. The distribution of intestinal flora, serum interleukin-8 (IL-8), IL-17, tumor necrosis factor-*α* (TNF-*α*) levels, recovery time of clinical symptoms, total effective rate of clinical symptoms, *H. pylori* eradication rate, and adverse reactions were observed.

**Results:**

2 weeks after treatment, the contents of *Bifidobacterium*, *Bacteroides*, and *Lactobacillus* in the intestinal tract of Group A decreased, while the amounts of *Enterococcus* and *Enterobacter* increased. In Group B, the contents of *Bifidobacterium*, *Bacteroides*, and *Lactobacillus* increased, while the amounts of *Enterococcus* and *Enterobacter* did not change significantly. Moreover, the trend of this flora change was still present at 4 weeks after treatment. Compared with Group A, Group B had lower IL-8, IL-17, and TNF-*α* levels, shorter recovery time of clinical symptoms, higher overall efficiency of clinical symptoms, and lower occurrence of adverse reactions. The eradication rate did not differ significantly between the two groups.

**Conclusion:**

BQT can lead to intestinal flora disorders in *cagA*+/*vacA s1m1 H. pylori* patients. *S. boulardii* can improve the distribution of intestinal flora, downregulate immune-inflammatory mediators, and modify clinical symptoms in patients.

## 1. Introduction

*H. pylori* is a spiral-shaped, microaerobic bacterium that colonizes the human gastric mucosa and is a class I carcinogen for gastric cancer. Although over 50% of the world population are infected with *H. pylori* [1], not all *H. pylori*-infected patients experience symptoms. The clinical outcome of patients with *H. pylori* infection varies depending on host genetic variables, environmental factors, and *H. pylori* virulence factors. *H. pylori* with the *cagA*+/*vacA s1m1* genotype is highly virulent and is the main causative strain of severe gastroduodenal disease [2]. CagA protein is the product of the *cag* pathogenicity island of *H. pylori* and is the most potent *H. pylori* virulence factor. A transgenic Drosophila model showed that CagA could cause dysbiosis of the intestinal microflora [3]. The *vacA* gene has several isoforms, of which s1m1 is the most virulent [4]. *vacA* encodes the protein VacA, a vacuolating cytotoxin secreted through the type V secretion system (T5SS) or autotransporter [4]. VacA can damage gastric mucosal cells through vacuolar degeneration of epithelial cells and can also modulate cytokine responses [5]. CagA and VacA can alter the gastric microbiota and immune phenotype in the *H. pylori-infected* stomach [6].

The confirmation of *H. pylori* infection is an indication for *H. pylori* eradication [7, 8]. The current consensus recommends BQT comprising proton pump inhibitor (PPI) and bismuth combined with two antimicrobial drugs for *H. pylori* eradication [7, 8]. The question of whether *H. pylori* eradication should be performed in older *H. pylori*-infected patients has been a widespread clinical concern [9]. However, there are few studies on *H. pylori* eradication in aged patients. The risk of adverse drug reactions to *H. pylori* eradication therapy increases in aged patients [10]. The treatment of *H. pylori* infection in this particular population should be evaluated comprehensively and managed individually [11]. *H. pylori* eradication is performed in patients who are *cagA*+/*vacA s1m1* to address the aged population who benefit more from eradication therapy. It is of great clinical significance to seek a safe and effective eradication therapy for such a population.

Probiotics are microorganisms with health benefits for the host. In recent years, the eradication rate of *H. pylori* has gradually decreased due to the emergence of bacterial drug resistance. Long-term and high-dose application of antibiotics can cause adverse effects such as intestinal dysbiosis and gastrointestinal dysfunction [12]. For some patients with gastrointestinal microbial instability, particularly older patients, the eradication of *H. pylori* should be done with more caution. From this perspective, probiotics address the problems of conventional therapies. Previous studies have shown that probiotics in eradication therapy can reduce adverse reactions and improve patient compliance [13]. *S. boulardii* is a fungal probiotic, and its action is not affected when taken with antibacterial drugs. *S. boulardii* can withstand local pressure, such as gastric acid, pepsin, and bile salt, so it maintains function in the gastrointestinal tract [14]. *S. boulardii* can also neutralize, blunt, and degrade pathogenic mycotoxins and participate in immune regulation [14, 15]. Compared to bacterial probiotics, its enormous body surface area allows better adhesion to pathogenic bacteria, resulting in superiority against *H. pylori* infection [15].

The clinical efficacy of *S. boulardii* in eradicating *H. pylori* remains controversial [16, 17]. In addition, previous studies have mainly focused on *S. boulardii* in terms of eradication rate and adverse reactions. This study is aimed at evaluating the effect of *S. boulardii* combined with BQT to eradicate *cagA*+/*vacA s1m1 H. pylori* on intestinal flora and immune-inflammatory mediators in older patients. We attempted to explore the feasibility of *S. boulardii* for the treatment of *H. pylori* from a new perspective.

## 2. Materials and Methods

### 2.1. Research Subjects

Patients aged between 60 and 70 years who attended the Department of Gastroenterology in our hospital from January 2021 to December 2021 were selected. This investigation was authorized by the Medical Ethics Committee of Xingtai People's Hospital. Informed consent was voluntarily signed by the patients or their families. This study follows the Helsinki Declaration.

Inclusion criteria: (a) age between 60 and 70 years; (b) ^14^C-urea breath test (^14^C − UBT) ≥ 100 dmp/mmoL CO_2_ or positive rapid urease test; (c) positive serum *H. pylori* IgG antibody typing test for both CagA and VacA (the *cagA*+/*vacA s1m1* genotype was identified by polymerase chain reaction (PCR)); (d) diagnosis of chronic nonatrophic gastritis by electronic gastroscopy; (e) patients with abdominal discomfort, acid reflux, abdominal distension, burning pain, belching, and other symptoms; and (f) initial *H. pylori* eradication treatment recipients. Exclusion criteria: (a) history of gastrointestinal surgery, tumor disease, tuberculosis, diabetes, rheumatic immune system diseases, chronic diarrhea, and constipation; (b) history of allergy to medicines and ingredients used in this research; (c) use of antibiotics, proton pump inhibitors (PPIs), bismuth, H_2_-receptor antagonists, microecological agents, hormones, and antifungal drugs within the last 3 months; (d) severe liver and kidney insufficiency; and (e) those who are unconscious, have psychiatric disease and communication disorders, and cannot cooperate with clinical treatment.

### 2.2. Methods

180 eligible patients were divided into Groups A and B according to the random number table method. Group A was treated with BQT, which involved 40 mg bid of pantoprazole sodium enteric tablets (Liaoning Novena Pharmaceutical Co., Ltd., State Drug Administration H20059067, specification 20 mg/tablet), 200 mg bid of colloidal bismuth pectin capsules (Shanxi Zhendong Ante Biopharmaceutical Co., Ltd., State Drug Administration H20058476, specification 0.1 g/capsule), 1000 mg bid of amoxicillin capsules (Kunming Baker Norton Pharmaceutical Co., Ltd., State Drug Administration H53021880, specification 0.5 g/tablet), and 500 mg bid of clarithromycin sustained-release tablets (Jiangsu Hengrui Pharmaceutical Co., Ltd., State Drug Administration H20031041, specification 0.5 g/tablet). The treatment duration was 2 weeks.

Group B was treated with the same BQT as Group A, combined with 500 mg bid of *S. boulardii* powder (France Baikota Pharmaceutical Factory, Import Drug Registration No. S20100086, specification 0.25 g/bag) for 2 weeks. Precautions: pantoprazole sodium enteric tablets, colloidal bismuth pectin capsules, and *S. boulardii* powder should be taken orally 0.5 hours before breakfast and dinner. Antibiotics should be taken immediately after breakfast and dinner. *S. boulardii* powder should not be taken with beverages above 50°C and should not be used with antifungal drugs to avoid affecting the activity of *S. boulardii* strains. Alcohol consumption is prohibited while taking antibiotics to prevent disulfiram-like reactions.

### 2.3. Observed Indicators

#### 2.3.1. Intestinal Flora

Fecal genomic extraction and PCR primer design: The contents of *Bifidobacterium*, *Bacteroides*, *Lactobacillus*, *Enterococcus*, and *Enterobacter* in the feces of the two groups were detected and compared. Fresh stool specimens (within 2 h after defecation) were collected from both groups before treatment and 2 and 4 weeks after treatment, saved in disposable sterile stool sampling boxes, and quickly stored in a low-temperature refrigerator at -80°C. Under sterile operation, 200 mg of stool was weighed from the center of the stool specimen. The TIANamp fecal DNA extraction kit was used for fecal sample genomic DNA extraction. After determining the concentration, it was stored at -20°C. Primers specific for the PCR target groups were designed according to the 16S rDNA sequences of *Bifidobacterium*, *Bacteroides*, *Lactobacillus*, *Enterococcus*, and *Enterobacter* [18, 19]. The specificity of the primer sequences was matched in the BLAST database (https://blast. ncbi. nlm. nih. gov/Blast. cgi). The relevant primer sequences are listed in Table 1.

PCR primer specificity assay: Five standard strains and fecal genomic DNA of healthy people were extracted for routine PCR amplification. The total volume of the reaction system was 25 *μ*L: 12.5 *μ*L of Premix Taq, 1 *μ*L of DNA template, 9.5 *μ*L of ddH_2_O, and 1 *μ*L each of upstream and downstream primers. Reaction conditions: 94°C for 5 min and annealing for 30 s; 72°C for 1 min, 30 cycles; 72°C for 10 min (see Table 1 for details). The PCR amplification samples were examined by 1.2% agarose gel electrophoresis. The results were observed under a gel imager. Only a single specific target band was seen, and no obvious nonspecific amplification was observed, indicating that the primers were specific and could be used for real-time PCR.

Real-time PCR: The kits used were from Tiangen Biochemical Technology (Beijing) Co. The plasmid standard substance was continuously diluted 10 times to obtain the standard substance with 10~107 series copies and was used as a template to construct the standard curve. The total volume of the reaction system was 20 *μ*L: 10 *μ*L of SYBR Green Master Mix, 1 *μ*L of upstream and downstream primers, 5 *μ*L of DNA template, and 3 *μ*L of ddH_2_O. Reaction conditions: 95°C for 5 min; 95°C for 10 s, annealing for 20 s (*Bifidobacterium* 57°C, *Bacteroides* 60°C, *Lactobacillus* 58°C, *Enterococcus* 52°C, and *Enterobacter* 58°C); and 72°C for 30 s, 45 cycles in total. At the conclusion of the reaction, a melting curve analysis was performed. After appropriately diluting fecal DNA, the reaction system and conditions were the same as above, and real-time PCR was conducted.

#### 2.3.2. Serum Levels of Inflammatory Factors

The serum levels of IL-8, IL-17, and TNF-*α* were measured and compared between the two groups. Three mL of fasting forearm venous blood was collected from the patients before treatment and 2 and 4 weeks after treatment. The serum was separated by centrifugation (Beijing Dinghaoyuan Technology Co., Ltd., model: NX-3), and the serum levels of IL-8, IL-17, and TNF-*α* were measured by enzyme-linked immunosorbent assay (ELISA). The instrument was supplied by Beijing Keyue Huacheng Technology Co., Ltd. An enzyme-linked immunosorbent assay kit was produced by TSZ Company (USA) and supplied by Shanghai Enzyme-linked Biological Technology Co., Ltd.

#### 2.3.3. Recovery Time of Clinical Symptoms

The main clinical symptoms in both groups were abdominal distension, heartburn, epigastric pain, and early satiety. The time required to ameliorate the above symptoms was evaluated and compared. The relevant indices were the time required for abdominal distension, heartburn, epigastric pain, and early satiety to alleviate.

#### 2.3.4. Total Efficiency of Clinical Symptoms

The total efficiency of the patients' symptoms was recorded and compared after treatment. The standards were listed below: the disappearance of gastric discomfort, bloating, epigastric pain, belching, acid reflux, heartburn, and early satiety was considered cured; significant improvement of gastric discomfort, bloating, epigastric pain, belching, acid reflux, heartburn, and early satiety was considered markedly effective; relief of gastric discomfort, bloating, epigastric pain, belching, acid reflux, heartburn, and early satiety was considered effective; no reduction of clinical symptoms was considered ineffective. Total effective rate = [(cured cases + markedly effective cases + effective cases)/total number of cases] × 100%.

#### 2.3.5. *H. pylori* Eradication Rate

After 4 weeks of withdrawal, the eradication rate of *H. pylori* was measured and compared between the two groups. ^14^C-UBT was detected in the two groups. Test results of ^14^C − UBT < 100 dmp/mmoL CO_2_ were considered *H. pylori*-negative, indicating successful eradication. Test results of ^14^C − UBT ≥ 100 dmp/mmoL CO_2_ were considered *H. pylori*-positive, indicating eradication failure. The *H. pylori* eradication rate was evaluated by per-protocol (PP) analysis and intention-to-treat (ITT) analysis to reduce the interference of analytical bias and loss of test information to improve the reliability of the conclusions.

#### 2.3.6. Adverse Reactions

The occurrence of adverse reactions, such as diarrhea, nausea, anorexia, and taste disorder, during the treatment period and 4 weeks after discontinuation of the drug was observed and recorded in the two groups. The overall incidence of adverse reactions was recorded in both groups.

### 2.4. Statistical Methods

SPSS 22.0 software was used for analyses and statistics. Normally distributed continuous variables are expressed as the mean ± standard deviation ($\bar{x}\pm s$), and a *t*-test was used for comparisons between two groups. Count data are expressed as percentages (%), and the *χ*^2^ test was used for comparisons between two groups. Differences were statistically significant when *P* < 0.05.

## 3. Results

### 3.1. General Information

**Figure 1:**
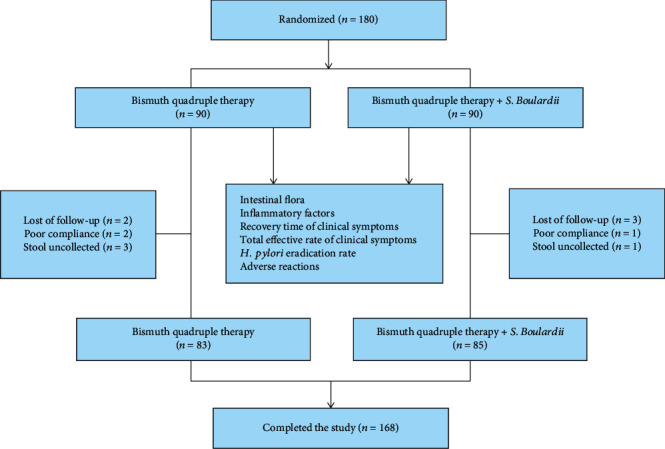
Subject flowchart.

Among 180 patients, 168 completed the experiment. In Group A, 2 patients were lost to follow-up, 2 patients had poor compliance, 3 patients did not receive stool collection, and 83 patients completed this study. In Group B, 3 patients were lost to follow-up, 1 patient had poor compliance, 1 patient did not have stool collection, and 85 patients completed this study (Figure 1). There were no meaningful differences in the general data, such as age, sex, body mass index (BMI), smoking history, drinking history, education level, exercise intensity, gastrointestinal symptoms, or Food Frequency Questionnaire (FFQ) evaluation, between the two groups (all *P* > 0.05) (Table 2).

### 3.2. Changes in Intestinal Flora Content at Different Time Points

**Figure 2:**
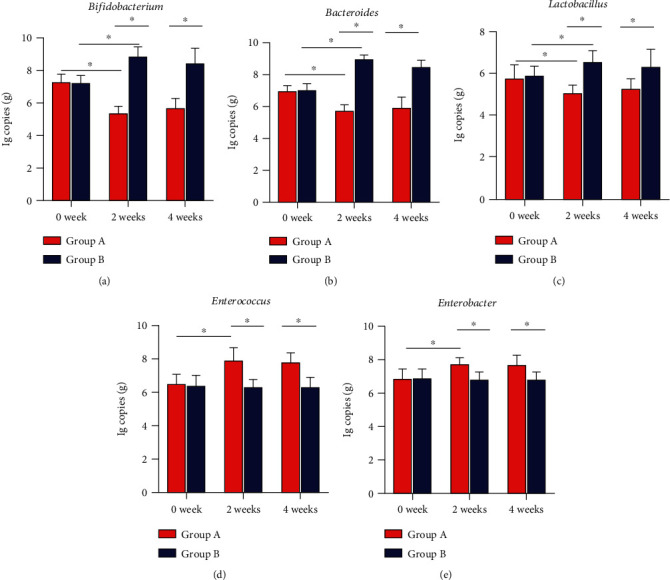
Intestinal flora content in the two groups of patients at different time points. (a) *Bifidobacterium*: at 2 weeks after therapy, the content of *Bifidobacterium* in Group A declined obviously (*P* < 0.05), while that in Group B raised (*P* < 0.05). The content of *Bifidobacterium* was significantly higher in Group B than in Group A (*P* < 0.05). This trend of change was still present at 4 weeks after therapy. (b) *Bacteroides*: at 2 weeks after therapy, the content of *Bacteroides* in Group A declined obviously (*P* < 0.05), while that in Group B raised (*P* < 0.05). The content of *Bacteroides* was significantly higher in Group B than in Group A (*P* < 0.05). This trend of change was still present at 4 weeks after therapy. (c) *Lactobacillus*: at 2 weeks after therapy, the content of *Lactobacillus* in Group A declined obviously (*P* < 0.05), while that in Group B raised (*P* < 0.05). The content of *Lactobacillus* was significantly higher in Group B than in Group A (*P* < 0.05). This trend of change was still present at 4 weeks after therapy. (d) *Enterococcus*: at 2 weeks after therapy, the content of *Enterococcus* in Group A raised obviously (*P* < 0.05), while the content of *Enterococcus* in Group B did not change significantly (*P* > 0.05). The content of *Enterococcus* was significantly less in Group B than in Group A (*P* < 0.05). This trend of change was still present at 4 weeks after therapy. (e) *Enterobacter*: at 2 weeks after therapy, the content of *Enterobacter* in Group A raised obviously (*P* < 0.05), while the content of *Enterobacter* in Group B did not change significantly (*P* > 0.05). The content of *Enterobacter* was significantly less in Group B than in Group A (*P* < 0.05). This trend of change was still present at 4 weeks after therapy. ^∗^*P* < 0.05.

There was no obvious distinction in the contents of intestinal *Bifidobacterium*, *Bacteroides*, *Lactobacillus*, *Enterococcus*, and *Enterobacter* between the two groups before therapy (*P* > 0.05). At 2 weeks after therapy, the contents of *Bifidobacterium*, *Bacteroides*, and *Lactobacillus* decreased (*P* < 0.05), and the contents of *Enterococcus* and *Enterobacter* increased (*P* < 0.05) in Group A compared with before therapy. The contents of *Bifidobacterium*, *Bacteroides*, and *Lactobacillus* increased (*P* < 0.05), and the contents of *Enterococcus* and *Enterobacter* did not change significantly (*P* = 0.365, *P* = 0.374) in Group B. At 2 weeks after therapy, Group B had more *Bifidobacterium*, *Bacteroides*, and *Lactobacillus* than Group A (*P* < 0.05) and less *Enterococcus* and *Enterobacter* than Group A (*P* < 0.05). In addition, the trend of this flora change was still present at 4 weeks after therapy (Figure 2).

### 3.3. Variation in Serum Inflammatory Factors at Different Time Points

**Figure 3:**
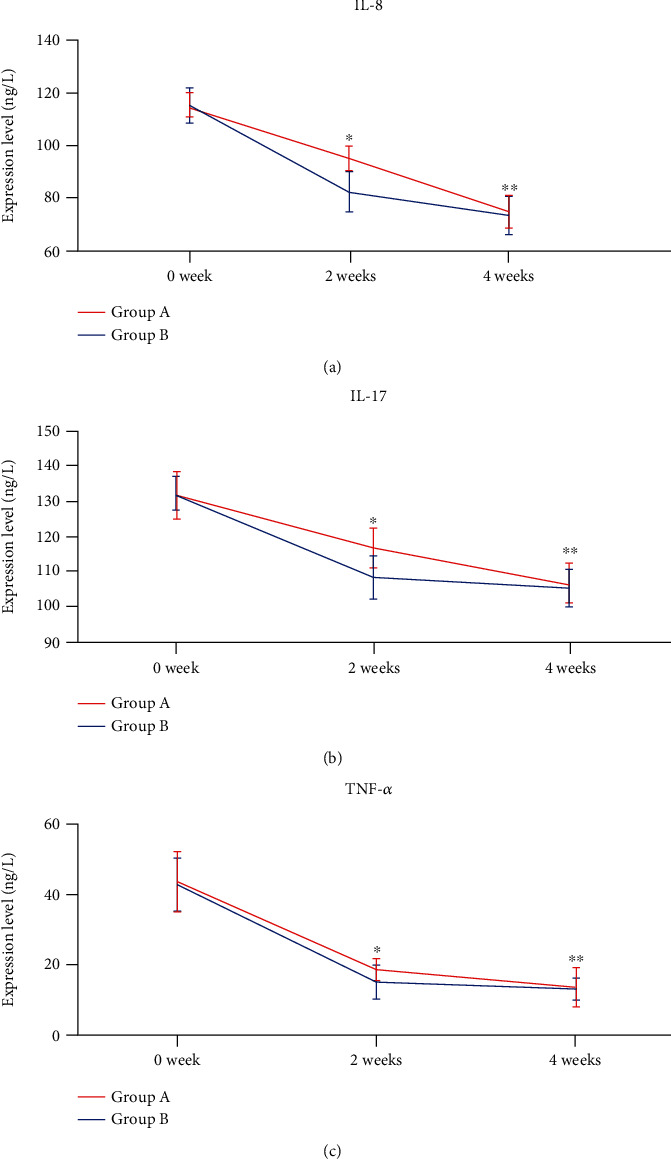
Serum inflammatory factors of patients at different time points. (a) IL-8: IL-8 levels in both groups were reduced significantly after 2 and 4 weeks of therapy. IL-8 levels in Group B were lower than those in Group A after 2 weeks of therapy (*P* < 0.05). However, there was no difference between the two groups after 4 weeks of therapy (*P* > 0.05). (b) IL-17: IL-17 levels in both groups were reduced significantly after 2 and 4 weeks of therapy. IL-17 levels in Group B were lower than those in Group A after 2 weeks of therapy (*P* < 0.05). However, there was no difference between the two groups after 4 weeks of therapy (*P* > 0.05). (c) TNF-*α*: TNF-*α* levels in both groups were reduced significantly after 2 and 4 weeks of therapy. TNF-*α* levels in Group B were lower than those in Group A after 2 weeks of therapy (*P* < 0.05). However, there was no difference between the two groups after 4 weeks of therapy (*P* > 0.05). ∗ indicates comparison before therapy (*P* < 0.05). ∗∗ indicates comparison 2 weeks after therapy (*P* < 0.05).

Before therapy, there was no statistically significant difference between the serum IL-8, IL-17, and TNF-*α* levels of the two groups of patients (*P* > 0.05). At 2 and 4 weeks after therapy, serum IL-8, IL-17, and TNF-*α* levels decreased significantly in both groups of patients (*P* < 0.05). At 2 weeks after therapy, serum IL-8, IL-17, and TNF-*α* levels in Group B were lower than those in Group A (*P* < 0.05), while there was no discrepancy between the two groups of patients at 4 weeks after therapy (*P* > 0.05) (Figure 3).

### 3.4. Recovery Time of Clinical Symptoms

**Figure 4:**
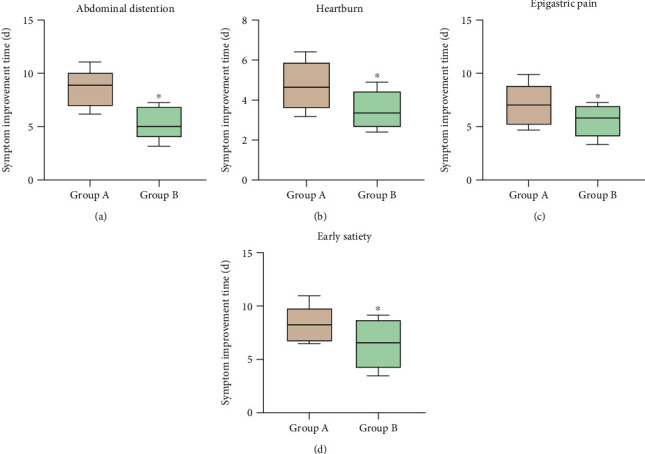
Recovery time of various clinical symptoms. (a) Recovery time for abdominal distension: In Group B, the recovery time for abdominal distension was significantly shorter than that in Group A (*P* < 0.05). (b) Recovery time for heartburn: In Group B, the recovery time for heartburn was significantly shorter than that in Group A (*P* < 0.05). (c) Recovery time for epigastric pain: In Group B, the recovery time for epigastric pain was significantly shorter than that in Group A (*P* < 0.05). (d) Recovery time for early satiety: In Group B, the recovery time for early satiety was significantly shorter than that in Group A (*P* < 0.05). ∗ denotes *P* < 0.05 when compared to Group A.

When comparing the recovery time for abdominal distension, heartburn, epigastric pain, and early satiety in the two groups, all the times were significantly shorter in Group B (*P* < 0.05) (Figure 4).

### 3.5. Total Effective Rate of Clinical Symptoms

At 2 weeks after treatment, the total effective rate of the two groups was compared. The total effective rate of patients in Group B was significantly higher than that in Group A (*P* < 0.05). Compared with Group A, patients in Group B had better symptom relief (Table 3).

### 3.6. *H. pylori* Eradication Rates

In Group A, 83 patients completed this study, and 64 patients were successfully eradicated. In Group B, 85 patients completed this study, and 70 patients were successfully eradicated. According to PP analysis, the eradication rates in Group A and Group B were 77.11% (64/83) and 82.35% (70/85), respectively. According to ITT analysis, the eradication rates in Group A and Group B were 71.11% (64/90) and 77.78% (70/90), respectively. None of the differences were statistically significant (Table 4).

### 3.7. Occurrence of Adverse Reactions

The occurrence of adverse reactions in both groups was diarrhea, nausea, loss of appetite, and taste disturbance. The incidence of adverse reactions in Group A and Group B was 25.30% and 8.24%, respectively, and the incidence of adverse reactions in Group B was lower than that in Group A. The difference was statistically significant (*χ*^2^ = 8.806, *P* = 0.003) (Table 5).

## 4. Discussion

*H. pylori* can stably colonize the strongly acidic environment, leading to persistent gastric mucosal damage through direct destruction and mediating inflammatory responses. The pathogenic mechanism of *H. pylori* is complex, and cytokines, colonization, virulence factors, free radicals, and other elements are involved. Virulence factors are important factors causing gastric mucosal lesions and even gastric mucosal carcinogenesis [20]. CagA can be transferred to cells under the action of the type IV secretion system to damage gastric mucosal epithelial cells directly and can also trigger DNA damage through oxidative stress [21, 22]. In addition, CagA can also activate the release of nuclear factor *κ*B (NF-*κ*B) and the inflammatory factor IL-8, resulting in atrophy, intestinal metaplasia, and carcinogenesis of gastric mucosal epithelial cells [23]. VacA is another major virulence factor of *H. pylori* that induces the body to produce immune-inflammatory factors, such as TNF-*α*, IL-12, and IL-8, inflicting mast cell degranulation [24]. *H. pylori* strains with the *cagA*+/*vacA s1m1* genotype can generate stronger cytokine responses and create more severe tissue inflammatory damage [25]. An effective *H. pylori* eradication regimen for older patients with *cagA*+/*vacA s1m1* chronic nonatrophic gastritis would better reflect individualized treatment.

*Bifidobacterium*, *Bacteroides*, and *Lactobacillus* are essential intestinal probiotic bacteria whose numbers and composition play a critical role in maintaining a healthy intestinal environment and improving immune system function [26, 27]. In particular, *Bacteroides* account for the major component of intestinal microbiota [26]. *Enterococcus* and *Enterobacter* are the important ESKAPE organisms highlighted by the World Health Organization as the leading causes of nosocomial and antibiotic-resistant infections in the last few decades, threatening public health [28]. In this study, alterations in the intestine of *Bifidobacterium*, *Bacteroides*, *Lactobacillus*, *Enterococcus*, and *Enterobacter* were examined before and 2 and 4 weeks after treatment. The study showed that the content of beneficial bacteria, such as *Bifidobacterium*, *Bacteroides*, and *Lactobacillus*, in the intestine of patients decreased 2 weeks after treatment with BQT. The contents of harmful bacteria, such as *Enterococcus* and *Enterobacter*, increased in patients. Furthermore, the trend of the bacteria listed above was still present at 4 weeks after treatment. The results showed that BQT could affect the intestinal flora structure of older patients, bringing about intestinal microecological imbalance, and the effect would still exist at 4 weeks after treatment. Intestinal bacteria are a huge microecological balance system. Its balance depends on various factors, such as species, number, and location of various floras. The floras coordinate and restrict each other to form an overall balanced microecosystem. The impact of *H. pylori* eradication therapy on intestinal microecology has received increasing attention [29]. The application of PPIs in *H. pylori* eradication regimens can reduce gastric acid secretion and decrease the clearance of exogenous pathogenic bacteria by gastric acid. In addition, the combined application of antibiotics can reduce the number of normal intestinal microbiota and increase the number of drug-resistant flora, leading to the imbalance of intestinal flora structure [30]. With increasing age, the number of intestinal core flora decreased, while the number of subdominant bacteria increased [31]. Therefore, the structure of the intestinal microecological system in aged patients undergoing drug treatment may be more likely to change [31]. Little is known about the changes in intestinal microecology in aged patients after BQT. Hsu et al. [32] reported that the relative abundance of *Firmicutes* and *Actinobacteria* in the feces of adults decreased significantly 2 weeks after reverse hybrid therapy. However, the content of *Proteobacteria* significantly increased, and the intestinal flora was in a disordered state. These changes in microbial flora did not persist but returned to the initial level at week 8 and week 48 [32]. Another study found that the level of *Bifidobacterium adolescentis* in the intestine decreased significantly after BQT, while the level of *Enterococcus faecium* rose. Moreover, *Enterococcus faecium* strains cultured in patients' stool after treatment were more resistant to antibiotics in vitro [33].

Probiotics can effectively prevent dysbacteriosis caused by *H. pylori* eradication therapy. Some scholars have used fecal microbial culture technology to analyze the effect of anti-*H. pylori* therapy on the intestinal flora. The microbial community in patients with concomitant therapy was significantly disturbed. The intestinal flora in patients receiving concomitant therapy combined with probiotics tended to be stable [34]. Tang et al. [35] used 16S rRNA high-throughput sequencing to analyze patients' fecal samples. The results showed that BQT led to the enrichment of some harmful bacterial groups in the intestine, such as *Shigella*, *Klebsiella*, and *Streptococcus*. Probiotic supplements could quickly restore these strains after treatment, increasing the abundance of *Bacillus* and *Lactobacillales* [35]. Traditional fecal bacterial culture methods have limitations such as high time consumption, high culture requirements, and many influencing factors. Real-time PCR and macrogenome sequencing technologies can preserve the complete genetic information of intestinal microorganisms, and the results are closer to the actual profile of the flora. It has obvious advantages in studying the functions of specific genes and metabolic pathways of microorganisms. However, macrogenome sequencing also has some limitations, such as the enormous computational effort to partition sequenced fragments, imperfect reference databases, large impact on the accuracy of algorithms, and expensive and complicated operation, which makes it difficult to be widely used for clinical testing. Real-time PCR overcomes the shortcomings of the high feature dimension of metagenomic sequencing, insensitivity to rare species, and weak biological background. The method is simple, easy to operate, has high sensitivity and specificity, and is suitable for large-scale clinical practice development. In this research, we applied real-time PCR to detect changes in intestinal flora in patients before and after BQT. After treatment of *S. boulardii* combined with BQT, there was no significant change in the contents of *Enterococcus* and *Enterobacter* in the intestines of patients, and the contents of *Bifidobacterium*, *Bacteroides*, and *Lactobacillus* increased. This result indicates that *S. boulardii* can effectively prevent the imbalance of intestinal flora caused by BQT in older patients.

*H. pylori* does not only cause a local inflammatory response in the gastric mucosa but also interacts with the host and mediates immune response, resulting in the upregulation of a series of inflammatory cytokines in the serum [36, 37]. A clinical study showed that serum TNF-*α* levels were significantly higher in *H. pylori*-positive patients than in *H. pylori*-negative patients. There were remarkable associations between serum TNF-*α* and IL-8 levels and the degree of mucosa chronic inflammation in patients [37]. The Th17/IL-17 immunoregulatory pathway is involved in *H. pylori* colonization and immune-inflammatory response [38]. The expression level of IL-17 was markedly elevated in the serum of *H. pylori*-infected individuals. IL-17 expression increased significantly with the severity of chronic gastritis [39]. Based on previous studies, we can find that *H. pylori* infection causes a series of severe inflammatory responses, resulting in elevated serum IL-8, IL-17, and TNF-*α* levels. This study showed that serum IL-8, IL-17, and TNF-*α* levels decreased significantly after treatment in both groups. The decrease in serum inflammatory factor levels was more pronounced in patients who underwent *S. boulardii* combined with BQT at 2 weeks after therapy. This result suggested that *S. boulardii* could downregulate *H. pylori*-mediated immune inflammatory responses and suppress serum IL-8, IL-17, and TNF-*α* levels. A study showed that *Lactobacillus gasseri* ATCC 33323 could depress the secretion of IL-8 in *H. pylori*-infected cell lines to ameliorate the inflammation caused by *H. pylori* [40]. *Lactobacillus salivarius* strains B37 and B60 produced diverse immune-regulating factors that restrain *H. pylori*-induced IL-8 production [41]. *Lactobacillus rhamnosus* 900 and *Lactobacillus paracasei* 915 suppress *H. pylori*-induced gastric epithelial cell production of IFN-*γ*, IL-12p70, IL-17A, and other inflammatory factors [42]. *S. boulardii* induced the production of specific immunoglobulin A (IgA) and secretory IgA, which repressed the secretion of inflammatory cytokines and exerted antibacterial infection effects [43]. Probiotics can change the host immune process and inhibit *H. pylori*-related inflammatory responses.

The clinical efficacy of probiotic-assisted eradication of *H. pylori* was inconsistent [16, 17, 44, 45]. One study showed that adding *S. boulardii* to sequential therapy increased eradication rate, reduced the occurrence of antibiotic-associated diarrhea and overall adverse events, and improved patient compliance [16]. Chang et al. [17] reported that *S. boulardii* combined with triple therapy neither increased eradication rates nor decreased adverse events. In our research, *S. boulardii* combined with BQT did not improve the eradication rate in *cagA*+/*vacA s1m1* patients. The different eradication outcomes may be related to whether the eradication regimen of *S. boulardii* combination is triple, sequential, or BQT. In addition, the type of antimicrobial drug in the eradication regimen and the duration of probiotic therapy may affect the clinical outcome. In this study, Group B had shorter recovery time of clinical symptoms, higher overall efficiency of clinical symptoms, and lower occurrence of adverse reactions. This is related to the downregulation of immune-inflammatory mediators and the modification of intestinal flora distribution by *S. boulardii*. Combined with the serum inflammatory factor results, we found that *S. boulardii* combined with QBT could better reduce serum IL-8, IL-17, and TNF-*α* levels. *S. boulardii* mediated the decrease in the levels of immune-inflammatory factors, effectively reducing the inflammatory response, thereby alleviating various clinical symptoms caused by *H. pylori*-associated gastritis and shortening the recovery time of symptoms. QBT contains two antibacterial drugs and PPI, with high drug doses and a long course of treatment. Some patients experienced a series of adverse reactions, such as nausea, diarrhea, and loss of appetite. The changes in intestinal microecology caused by QBT were involved in the occurrence of these adverse reactions. *S. boulardii* can modify the distribution of intestinal flora, thus effectively reducing adverse effects.

However, this study only observed intestinal flora changes at 2 and 4 weeks after QBT. The long-term effects of eradication therapy on gut microecology need to be studied in-depth. In addition, the effectiveness of probiotic-assisted eradication of *H. pylori* strains of different genotypes requires further investigation. It is unclear whether the current decline in the *H. pylori* eradication rate and the emergence of multiple drug resistance are associated with secondary dysbacteriosis due to eradication therapy. Future multicenter randomized controlled studies with large samples are needed to clarify the association between gastric microbiota profile and *H. pylori* eradication therapy to effectively enhance eradication efficacy.

## 5. Conclusions

BQT can lead to intestinal flora disorders in *cagA*+/*vacA s1m1 H. pylori* patients. *S. boulardii* supplementation did not improve eradication rates. However, *S. boulardii* can improve the distribution of intestinal flora, downregulate immune-inflammatory mediators, and modify clinical symptoms. This study has a certain reference value for the eradication of *H. pylori* in older patients.

## Figures and Tables

**Table 1:** Genus-specific primer information.

Bacterial genus	Primer sequences	Annealing temperature	Amplified fragments (bp)
*Bifidobacterium*	F: 5′-CTCCTGGAA ACGGGTGG-3′ R: 5′-GGTGTTCTTCCCGATATCTACA-3′	57	549~563
*Bacteroides*	F: 5′-ATAGCCTTTCGAAAGRAAGAT-3′ R: 5′-CCAGTATCAACTGCAATTTTA-3′	50	501
*Lactobacillus*	F: 5′-AGCAGTAGGGAATCTTCCA-3′ R: 5′-CACCGCTACACATGGAG-3′	53	341
*Enterococcus*	F: 5′-CCCTTATTGTTAGTTGCCATCATT-3′ R: 5′-ACTCGTTGTACTTCCCATTGT-3′	57	140
*Enterobacter*	F: 5′-GTTAATACCTTTGCTCATTGA-3′ R: 5′-ACCAGGGTATCTAATCCTGTT-3′	55	340

**Table 2:** General information of patients in both groups.

Group	Group A (*n* = 90)	Group B (*n* = 90)	*P* value
Gender			0.549
Male	52 (57.78)	48 (53.33)	
Female	38 (42.22)	42 (46.67)	
Age (yr)^a^	66.2 ± 2.51	65.6 ± 3.32	0.159
BMI (kg/m^2^)^a^	24.26 ± 2.35	23.89 ± 1.96	0.253
Smoking	19 (21.11)	16 (17.78)	0.572
Alcohol	24 (26.67)	28 (31.11)	0.511
Years of education (yr)^a^	10.23 ± 1.95	9.86 ± 1.82	0.190
Patient's family residence			0.541
Urban	53 (58.89)	57 (63.33)	
Rural	37 (41.11)	33 (36.67)	
Job			0.627
Manual labor	26 (28.89)	29 (32.22)	
Mental labor	64 (71.11)	61 (67.78)	
Physical activity IPAQ			0.647
Low	23 (25.56)	28 (31.11)	
Moderate	58 (64.44)	52 (57.78)	
High	9 (10.00)	10 (11.11)	
Food Frequency Questionnaire (FFQ)			
Calories (kcal)^a^	2136.75 ± 326.29	2098.43 ± 416.38	0.493
Protein (g)^a^	75.82 ± 1.96	76.09 ± 2.34	0.403
Fat (g)^a^	42.89 ± 2.15	43.12 ± 1.97	0.455
Carbohydrates (g)^a^	237.45 ± 13.49	240.58 ± 12.37	1.106
Gastrointestinal symptoms			0.895
Mild	20 (22.22)	18 (20.00)	
Moderate	46 (51.11)	49 (54.44)	
Severe	24 (26.67)	23 (25.56)	
Yogurt			0.661
Yes	11 (12.22)	13 (14.44)	
No	79 (87.78)	77 (85.56)	
History of hypertension			0.530
Yes	57 (63.33)	61 (67.78)	
No	33 (36.67)	29 (32.22)	
History of hyperlipidemia			0.615
Yes	26 (28.89)	23 (25.56)	
No	64 (71.11)	67 (74.44)	

^a^Data are presented as the mean ± standard deviation (SD).

**Table 3:** Total effective rate of patients in both groups.

Classification	Group A (*n* = 83)	Group B (*n* = 85)	*χ* ^2^ value	*P* value
Cured	29 (34.94)	32 (37.65)	—	—
Markedly effective	22 (26.51)	24 (28.24)	—	—
Effective	14 (16.87)	21 (24.71)	—	—
Ineffective	18 (21.69)	8 (9.41)	—	—
Total effective rate (%)	65 (78.31)	77 (90.59)	4.837	0.028

**Table 4:** The eradication rate of *H. pylori.*

Classification	Group A	Group B	*χ* ^2^ value	*P* value
Total number (*n*)	90	90	—	—
Complete number (*n*)	83	85	—	—
Successful eradication (*n*)	64	70	—	—
PP eradication rate (%)	64/83 (77.11)	70/85 (82.35)	0.716	0.398
ITT eradication rate (%)	64/90 (71.11)	70/90 (77.78)	1.051	0.305

**Table 5:** Incidence of adverse reactions.

Classification	Group A (*n* = 83)	Group B (*n* = 85)	*χ* ^2^ value	*P* value
Diarrhea	15 (18.07)	3 (3.53)	—	—
Nausea	2 (2.41)	2 (2.35)	—	—
Anorexia	3 (3.61)	1 (1.18)	—	—
Taste disorder	1 (1.20)	1 (1.18)	—	—
Incidence of adverse reactions (%)	21/83 (25.30)	7/85 (8.24)	8.806	0.003

## Data Availability

The authors confirm that the data supporting the findings of this study are available within the article.
